# Modification of *in situ* Biofilm Formation on Titanium by a Hydroxyapatite Nanoparticle-Based Solution

**DOI:** 10.3389/fbioe.2020.598311

**Published:** 2020-12-04

**Authors:** Cíntia M. G. Nobre, Norbert Pütz, Belinda König, Stefan Rupf, Matthias Hannig

**Affiliations:** Clinic of Operative Dentistry, Periodontology and Preventive Dentistry, Saarland University Hospital, Homburg, Germany

**Keywords:** biomaterials, oral biofilm, implantology, titanium, hydroxyapatite

## Abstract

Oral biofilms play an essential role on peri-implant disease development. Synthetic hydroxyapatite nanoparticles (nHAP) are a bioinspired material that has structural and functional similarities to dental enamel apatite and may provide preventive properties against biofilm formation. This study aimed to investigate the effects of an experimental nHAP solution on biofilm formation on polished and non-polished titanium under oral conditions. Five volunteers carried maxillary splints with non-polished and polished titanium and followed a 48 h rinsing protocol with the proposed nHAP solution, and with chlorhexidine 0.2% (CHX) and water, as controls. Samples were analyzed by fluorescence microscopy (FM), scanning electron microscopy (SEM) and transmission electron microscopy (TEM). FM showed a significant reduction of biofilms on polished samples treated with nHAP (*p* = 0.0485) compared with water, without differences between nHAP and CHX (*p* > 0.9999). Analyzing biofilm viability, polished samples rinsed with nHAP showed significantly fewer dead bacteria than CHX (*p* = 0.0079), but there was no significant difference in viability between polished samples rinsed with water and nHAP (*p* = 0.9268). A significantly higher biofilm coverage was observed on the non-polished surfaces compared to the polished surfaces when nHAP was applied (*p* = 0.0317). This difference between polished and non-polished surfaces was not significant when water (*p* = 0.1587) or CHX (*p* = 0.3413) rinsing were applied. SEM and TEM analysis supported the FM findings, that polished samples rinsed with nHAP presented fewer biofilm coverage compared to samples rinsed with water. In conclusion, the nHAP solution reduced the biofilm formation on polished Ti surfaces without altering bacterial viability, providing a novel approach for the management of biofilm formation on biomaterials.

## Introduction

Dental implants are one of the greatest advancements in dentistry. They are a well-established and predictable method for partial and total oral rehabilitation. Titanium (Ti) is the most common material used for dental and medical implants due to its biocompatibility and mechanical properties (Muddugangadhar et al., 2015). However, there are still multiple factors that can affect the clinical success of implants, including the risk of bacterial colonization around the titanium device (Veerachamy et al., 2014). Biofilm formation usually occurs after Ti implants exposure to the oral cavity and may lead to persistent infection, playing an essential role for peri-implant disease development (Fürst et al., 2007; Veerachamy et al., 2014).

Periimplantitis is an inflammatory disease that affects the soft and hard tissue around an implant. It starts with pellicle formation followed by bacteria adhesion to the titanium surface, leading to biofilm formation. Once a mature multi-layered biofilm is formed, the bacteria are extremely resistant to conventional antimicrobial therapies and immune system lines of defense and may lead to an inflammatory response. The inflammatory process starts on the soft tissue surrounding the dental implant (peri-implant mucositis) and can evolve causing progressive loss of supporting bone, leading to implant failure (Smeets et al., 2014; Schwarz et al., 2018).

Until the present date, chlorhexidine (CHX) is still the first-choice adjunct solution for prevention of dental biofilm formation. It is widely used as a broad-spectrum antiseptic, being the gold standard in dentistry (James et al., 2017). However, despite its antimicrobial effect, CHX is not recommended for long-term use, due to various adverse effects such as teeth staining, oral mucosal erosion, and transient taste disturbance (James et al., 2017). Therefore, to achieve reasonable biofilm control and less adverse effects as possible, the search for new biomimetic materials is of utmost importance.

Research concerning the application of hydroxyapatite nanoparticles (nHAP) in dentistry has increased within the past few years. Hydroxyapatite is a calcium phosphate ceramic and the main mineral component of the tooth. Synthetic nHAP has structural and functional similarities to dental enamel apatite. It can mimic the enamel crystallites, which are the smallest building units of dental enamel, constituting the enamel prisms (Sakae et al., 2011; Kensche et al., 2017). As a bioinspired material, hydroxyapatite is non-toxic and non-immunogenic when applied in adequate doses (Epple, 2018). Kensche et al. (2017) observed that a mouthwash containing hydroxyapatite particles could reduce the number of adherent bacteria on enamel specimens, having comparable effects to chlorhexidine. Recently, Nobre et al. (2020) observed that hydroxyapatite nanoparticles could adhere not only to enamel but also to dental material surfaces, such as titanium, under oral conditions. Thus, nHAP may have preventive properties against biofilm formation also on titanium.

In this study, an *in situ* experimental model has been applied due to its suitability to reproduce the intraoral conditions and to understand the influence of nHAP on oral biofilm formation (Hannig et al., 2007; Hannig and Hannig, 2009).

The objective of this *in situ* study was to investigate the effects of a biomimetic pure nanohydroxyapatite solution on biofilm formation on titanium. The hypothesis was that this bioinspired nHAP solution would reduce biofilm formation on Ti surfaces.

## Materials and Methods

### Subjects

This *in situ* experiment evaluated the biofilm formation on titanium in five healthy volunteers aged between 28 and 35 years, who are members of the laboratory staff of the Clinic of Operative Dentistry, Periodontology and Preventive Dentistry, Saarland University. The subjects had to fulfill the following inclusion criteria: good oral health with no signs of gingivitis, caries or unphysiologically salivary flow rate; no systemic diseases; no use of antibiotics or any kind of periodontal treatment within the past 6 months; non-smoker; not pregnant or breastfeeding and absence of orthodontic appliances, confirmed after an intraoral examination and a questionnaire.

The study protocol was approved by the Medical Ethics Committee of the Medical Association of Saarland, Germany (no 283/2009–2016). Informed written consent concerning the participation in the study was obtained from all subjects.

### Titanium Samples

Titanium discs (5 mm diameter; 1 mm height) with micro-structured surfaces and treated with the sandblasted and acid-etched (SLA) technique (Ra = 2 μm, grade 2), were obtained from Dentsply Implant Systems (Dentsply Sirona, Bensheim, Germany). Half of the samples remained unpolished, while the other half was polished by wet grinding with abrasive paper (800 to 4000 grit). To remove the resulting smear layer and for disinfection purpose, Ti discs were immersed in isopropanol (70%) for 10 min, followed by ultrasonic bath in distilled water.

### Tested Solution

Hydroxyapatite nanoparticles (Kalident powder 100 nm) were supplied by Kalichem Srl, Italy. The containing nHAP test solution was prepared mixing 0.5 g powder in 10 ml bidistilled water. Chlorhexidine mouthwash [0.2% (w/v) chlorhexidine digluconate in 7% (v/v) ethanol (Saarland University Hospital Pharmacy, Homburg, Germany] and distilled water rinse (10 ml) served as positive and negative controls, respectively. The subjects used different rinsing solutions in different weeks to avoid interferences between test and control solutions, preventing a possible cross-over effect. The first solution used by each volunteer was the water control. One week later, the nHAP test solution was introduced. The volunteers performed a final rinse with the CHX control solution after an additional 2 weeks clearance period.

### Oral Exposure

**FIGURE 1:**
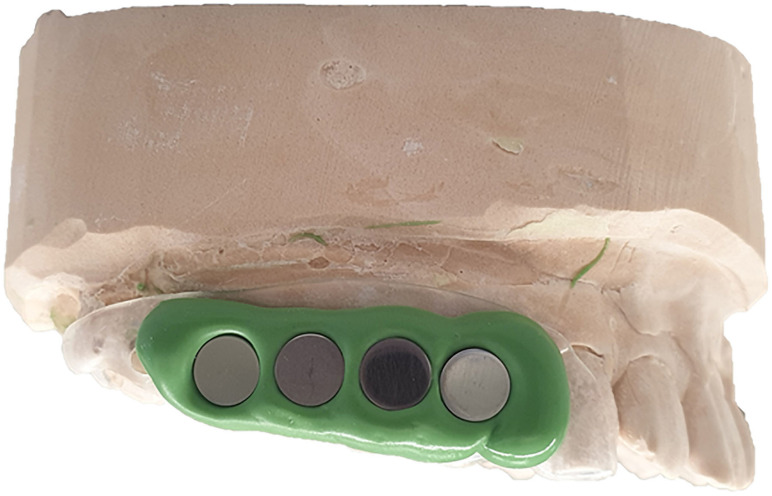
Splint with mounted titanium specimens.

**FIGURE 2:**
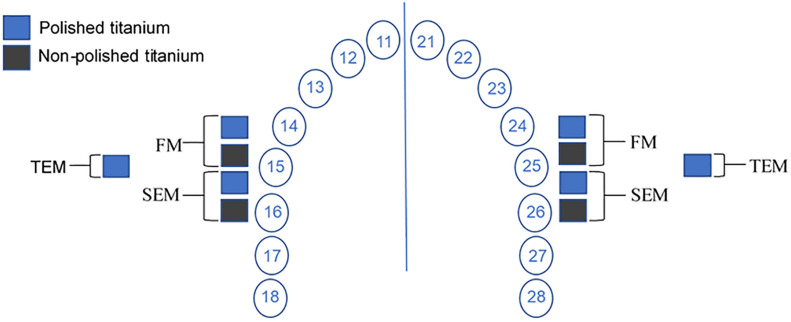
Schematic drawing of sample location in the upper jaw splint and the subsequent analyses: fluorescence microscopy (FM), scanning electron microscopy (SEM) and transmission electron microscopy (TEM). The samples for TEM analysis were attached posteriorly to other splints in the same volunteers. This arrangement was reproduced for each one of the three tested solutions (hydroxyapatite nanoparticles, chlorhexidine and water).

Titanium samples were mounted in customized maxillary splints to evaluate *in situ* biofilm formation (Figure 1). They were prepared from 1.5 mm thick methacrylate foils, extending from premolars to the second molar. Perforations in the buccal aspects of the splints were prepared to fix the polyvinyl siloxane impression material, in which the Ti discs were placed. Initially, four samples (2 polished and 2 non-polished) were mounted in each upper quadrant, totalizing eight samples per volunteer for each rinsing solution (Figures 1, 2).

Before intraoral exposure of the splints, the volunteers brushed their teeth without toothpaste and rinsed with tap water only to avoid possible interferences from the compounds of the toothpaste. Each volunteer performed the rinse with 10 ml of the nHAP-based solution during 30 s four times during a period of 48 h. The mouthwashes were performed 3 min, 12, 24, and 36 h after the insertion of the splint. During this experiment, the participants did not use toothpaste or any other kind of mouthwash for oral hygiene purpose. Volunteers also took off their splints during meals, brushed their teeth without toothpaste after eating to avoid any interference from the toothpaste compounds, and placed the splints again after 10 min. Splints were stored in a plastic box at 100% humidity and room temperature.

After 48 h, Ti discs were removed and immediately rinsed with distilled running water to remove non-adsorbed salivary components. Then, two samples (one each side) were prepared for fluorescence microscopy (FM) and another two (one each side) for scanning electron microscopy (SEM). The same protocol was used to prepare two additional polished samples (one each side) for transmission electron microscopy (TEM) (Figure 2).

### BacLight Viability Assay

The BacLight viability assay differentiates living from dead bacteria based on two nucleic acid stains: SYTO 9 and propidium iodide. While the first one stains green all bacteria (with intact or damaged membranes), the last one stains red cells with compromised membranes. When mixed, propidium iodide reduces SYTO 9 fluorescence, enabling a viability evaluation between live and dead bacteria (Stiefel et al., 2015).

The BacLight viability assay was carried out at room temperature in a 6-well-plate. One micro-liter SYTO 9 and 1 μl PI were mixed in 1 ml saline solution (0.9% NaCl). This staining solution was vortexed before use. Ti samples were covered with 10 μl staining solution and left 10 min in a dark chamber. Subsequently, Ti discs were washed in saline solution two times, fixed to a glass slide and mounted in BacLight oil.

### Fluorescence Microscopy

The detection of bacteria and their viability was conducted with FM at 1000-fold magnification (Axioskop II, ZEISS MicroImaging GmbH, Göttingen, Germany), using AxioVision 4.8 (Carl Zeiss MicroImaging GmbH, Göttingen, Germany) for image processing. Nine random representative pictures per sample were taken. Bacteria coverage was evaluated with Sefexa Image Segmentation Tool. For live and dead cell correlation, a scoring system was used.

#### Scoring System

The viability correlation between live and dead cells observed in the biofilm on the titanium discs samples were assessed and scored by two calibrated examiners (90% agreement rate) and it was based on the following scoring system table (Table 1) described by Rupf et al. (2012).

**TABLE 1:** Scoring system for the assessment of biofilm viability detected with the BacLight viability assay.

Score	Description
*1*	Mainly red fluorescence; ratio between red and green fluorescence 90:10 and higher.
*2*	More red fluorescence; ratio between red and green fluorescence 75:25 and higher.
*3*	Ratio between red and green fluorescence 50:50.
*4*	More green fluorescence; ratio between red and green fluorescence 25:75 and lower.
*5*	Mainly green fluorescence; ratio between red and green fluorescence 10:90 and lower.

### Scanning Electron Microscopy

Titanium samples were also prepared for an SEM analysis to investigate biofilm coverage and to detect adherent nHAP particles. After 48 h oral exposure, Ti discs were washed with sterile water. After washing, samples were fixated with 1 ml 2% Glutaraldehyde in 0,1 M cacodylate buffer during 1 h at 4°C. Next, samples were washed five times, 10 min each, with 1 ml of cacodylate buffer. A series of ethanol dehydration followed this procedure. Samples were immersed in various ethanol solutions accordingly: ethanol 50% (2 × 10 min), ethanol 70% (1 × 5 min), ethanol 80% (1 × 5 min), ethanol 90% (1 × 5 min) and ethanol 100% (2 × 10 min). Finally, the samples were dried in 1,1,1,3,3,3-hexamethyldisilazane (HMDS, Acros Organics, Geel, Belgium). HMDS was vaporized at room temperature in a clean bench. Finally, Ti discs were left to airdry overnight at room temperature in the air chamber. Samples were attached to aluminum stubs, sputtered and coated with carbon. SEM and EDX evaluations were made in an XL30 ESEM FEG (FEI, Eindhoven, Netherlands) at 5 and 10 kV, consecutively, at up to 20,000-fold magnification.

### Transmission Electron Microscopy

To better understand the test solution effects on biofilm formation, it was also decided to investigate the ultrastructural characteristics of the obtained biofilm by TEM. Immediately after volunteers took off their splints, Ti discs were washed with sterile water to remove not adhered bacteria. Then, the specimens were placed in 1,5 ml tubes with 1 ml 1% Glutaraldehyde fixing solution at 4°C during 1 h. After primary fixation, samples were washed with cacodylate buffer 0.1 M 4 times, 10 min each, and stored at 4°C in cacodylate buffer. Ti discs were subjected to a secondary fixation in osmium tetroxide during 1 h in a dark chamber at room temperature, followed by five times 10 min wash in distilled water and immersion in 30% ethanol overnight.

Following the TEM preparation procedures, dehydration was performed at room temperature. Samples passed through series of 50% (2 × 10 min), 70% (2 × 20 min), 90% (2 × 30 min), and 100% (2 × 30 min) ethanol. Finally, Ti discs were further immersed in 100% acetone two times, 30 min each, and stored overnight in an acetone/Araldite (Electron Microscopy Sciences, United States) mixture plus 3% accelerator (mixture A) at room temperature. On the following day, mixture A was poured out, and a second mixture, mixture B was prepared (Araldite mixture with 2% accelerator). Samples were left again overnight in mixture B at room temperature in the air chamber. Next, a new mixture B was used to fill half of the embedding forms. Notes with identification number were placed at the bottom side. Ti discs were placed, and the embedding forms was filled until the top again with mixture B. Then, samples were incubated for polymerization for 48 h at 65°C. After polymerization, Ti was removed by treatment with hydrofluoric acid (5%) during 48 h, and the specimens were re-embedded in Araldite.

Finally, samples were cut in ultra-thin sections in an ultramicrotome with a diamond knife (Leica EM UC7, Germany) and mounted on Pioloform-coated copper grids and contrasted with aqueous solutions of uranyl acetate and lead citrate at room temperature. After an intensive wash with distilled water, biofilm inner and out layers could be then analyzed with a TEM Tecnai 12 BioTwin (FEI, Eindhoven, Netherlands) under a magnification up to 100.000-fold.

### Statistics

The mean values were analyzed using GraphPad Prism 6. Mann-Whitney test was performed to evaluate the differences between polished and non-polished titanium samples for each solution used, and Kruskal-Wallis test with Dunn’s correction for multiple comparisons test to access the differences between all polished samples in all solutions. Statistical significance was considered for *p* < 0.05.

## Results

### Fluorescence Microscopy BacLight Assay

Biofilms were formed within 48 h in all samples, regardless of the solution used. However, there were variations concerning the quantity and viability of biofilms that covered at the Ti surfaces and the bacterial viability.

#### Biofilm Coverage

**FIGURE 3:**
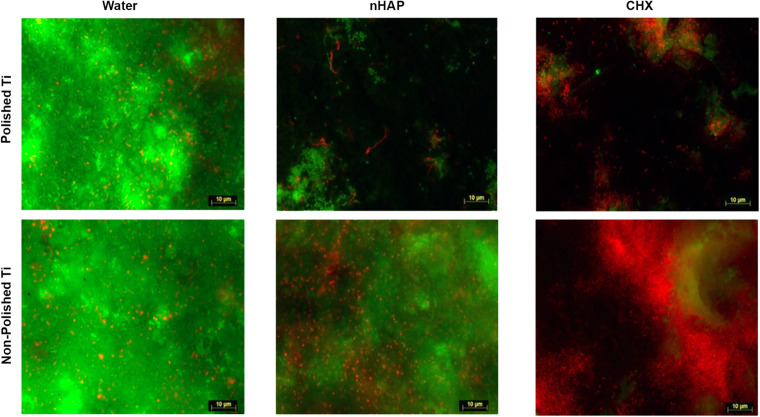
Fluorescence microscopic investigation of Live/Dead stained biofilm allows bacterial coverage visualization and differentiation between live (green) and dead (red) bacteria. After 48 h, non-polished samples presented significantly higher amounts of biofilms than polished samples. While water negative controls appeared densely covered with live bacteria, samples rinsed with chlorhexidine 0.2% and hydroxyapatite nanoparticles presented less biofilm formation.

**FIGURE 4:**
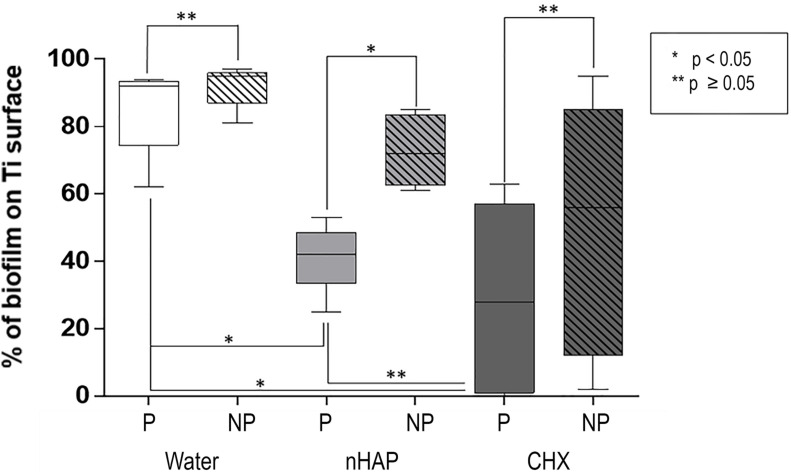
Biofilm coverage of the polished samples (P): chlorhexidine 0.2% (CHX) and hydroxyapatite nanoparticles-based solution (nHAP) reduced the bacterial adherence on Ti discs surfaces compared to water. There was no significant difference between the gold standard CHX and the nHAP test solution. Comparing polished and non-polished samples (NP), the only significant difference was between P and NP samples when nHAP solution was used.

As shown in Figures 3, 4, samples rinsed with water presented a thick biofilm layer, covering the majority of the Ti surfaces after 48 h. There was no significant difference between polished and non-polished surfaces when rinsed with water (*p* = 0.1587) or with CHX (*p* = 0.3413). However, the difference was significant between polished and non-polished samples rinsed with nHAP (*p* = 0.0317). Furthermore, another predictable result was the significantly lower biofilm coverage after treatment with CHX 0.2% when compared with the water rinse (*p* = 0.0215) on polished samples. Similar results were achieved comparing nHAP test solution and water (*p* = 0.0485) but with no significant difference between nHAP and CHX (*p* > 0.9999).

#### Biofilm Viability

**FIGURE 5:**
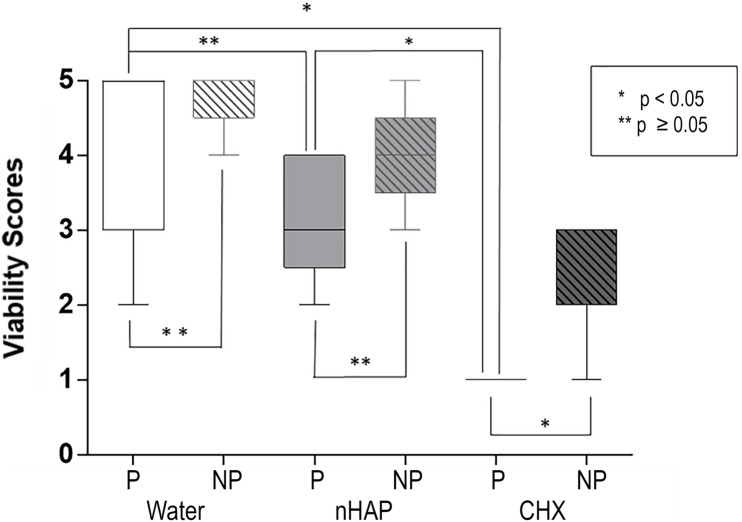
Biofilm viability: chlorhexidine (CHX) significantly reduced the number of live bacteria compared to the negative control on polished (P, *p* = 0.0037) and on non-polished (NP, *p* = 0.0079) samples. Non-significant reduction of viability between hydroxyapatite nanoparticles-based solution (nHAP) and water samples was present on P (*p* = 0.9268) and NP (*p* = 0.1667) titanium discs.

Scoring was performed to analyze the biofilm viability (Figure 5 and Table 1). There was no significant difference between polished and non-polished samples rinsed with water or nHAP concerning the bacteria viability, presenting a majority of live bacteria. However, it could be observed significantly more live bacteria on non-polished samples rinsed CHX than on polished samples rinsed with the same solution (*p* = 0.0476). Regarding the polished samples, significantly more live bacteria could be seen after water rinsing, when compared with CHX (*p* = 0.0037). However, no significant difference between polished samples rinsed with water and with nHAP could be detected (*p* = 0.9268). Samples rinsed with CHX showed a significant reduction in the number of vital bacteria compared with nHAP rinsed samples (*p* = 0.0079).

### Scanning Electron Microscopic Analysis

**FIGURE 6:**
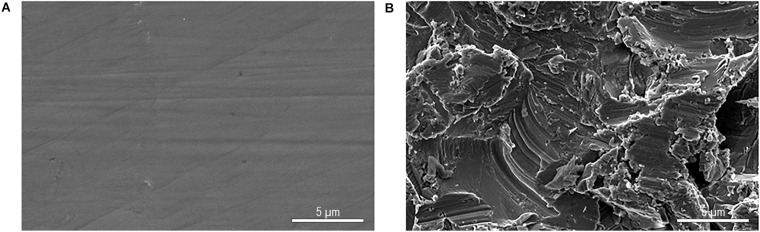
SEM figures at 5,000-fold magnification from original titanium specimens not exposed to the oral cavity polished by wet grinding with abrasive paper from 800 to 4000 grit **(A)** and without polishing **(B)**.

**FIGURE 7:**
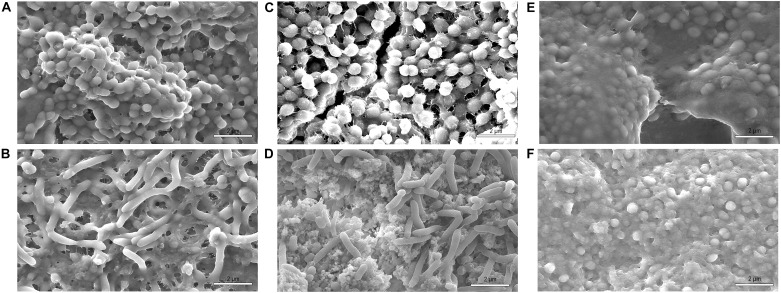
SEM at 10,000-fold magnification of non-polished samples shows a mature biofilm with cocci and rod-shaped bacteria species after 48 h of *in situ* intraoral exposure in volunteer 1 **(A,C,E)** and volunteer 2 **(B,D,F)**. Samples rinsed with water **(A,B)**, hydroxyapatite nanoparticles-based solution **(C,D)** and chlorhexidine **(E,F)**. In samples rinsed with chlorhexidine most of the bacteria found were cocci.

Scanning electron microscopy analyses were performed on two types of surfaces: polished and non-polished titanium (Figure 6). The 48-hour biofilm presented on titanium surfaces samples from healthy volunteers were associated predominantly with coccoid or rod-shaped bacteria. These bacteria were distributed randomly on the titanium surfaces as individual bacteria or colonies (Figure 7). These observations were independent of the surface type.

**FIGURE 8:**
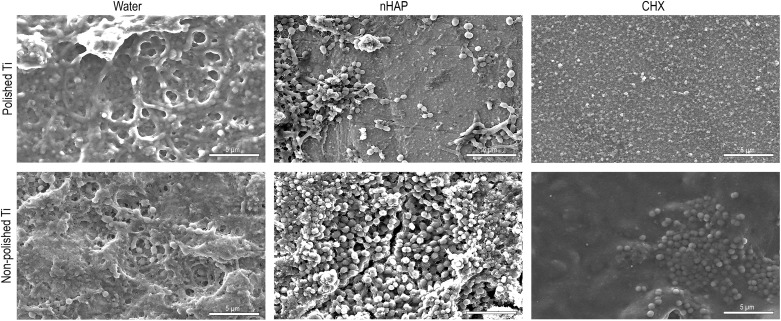
SEM images at 5,000-fold magnification of polished and non-polished titanium (Ti) surfaces after 48 h intraoral exposure and rinsing with water, hydroxyapatite nanoparticles-based (nHAP) solution, and chlorhexidine (CHX).

Independent of the surface topography, Ti samples from the water control had thicker biofilm covering compared to nHAP and CHX samples (Figure 8). Polished samples treated with hydroxyapatite or chlorhexidine solutions presented areas with thin biofilm layers and areas without biofilm (Figure 8). Therefore, SEM analysis corroborated the FM results, indicating that the nHAP rinsing solution reduced the amount of mature biofilm formed on polished Ti surfaces.

**FIGURE 9:**
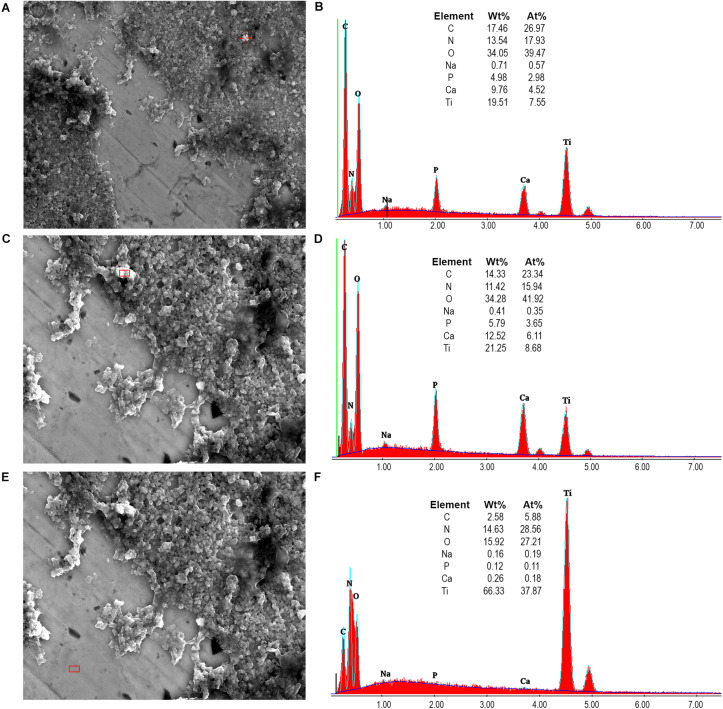
SEM images at 5,000-fold **(A)** and 10,000-fold **(C,E)** magnifications allowed the visualization of hydroxyapatite nanoparticles which accumulated at the titanium surfaces, with sizes varying from 90 nm to 500 nm. Higher values of P and Ca detected by the EDX analysis **(B,D)** confirmed the presence of hydroxyapatite nanoparticles. Low percentages of P and Ca, as well as the high Ti percentage on panel **(F)**, corroborated to these results as a negative control, showing the presence of only titanium in the demarcated area on panel **(E)**.

Furthermore, Figure 9 shows that hydroxyapatite nanoparticles and aggregates could be detected randomly on the pellicle surface after 48 h of intraoral exposure when rinsing with the nHAP test solution. Energy-dispersive X-ray spectroscopy (EDX) analysis endorsed the SEM results, confirming the presence of hydroxyapatite through elements quantification.

### Transmission Electron Microscopic Analysis

**FIGURE 10:**
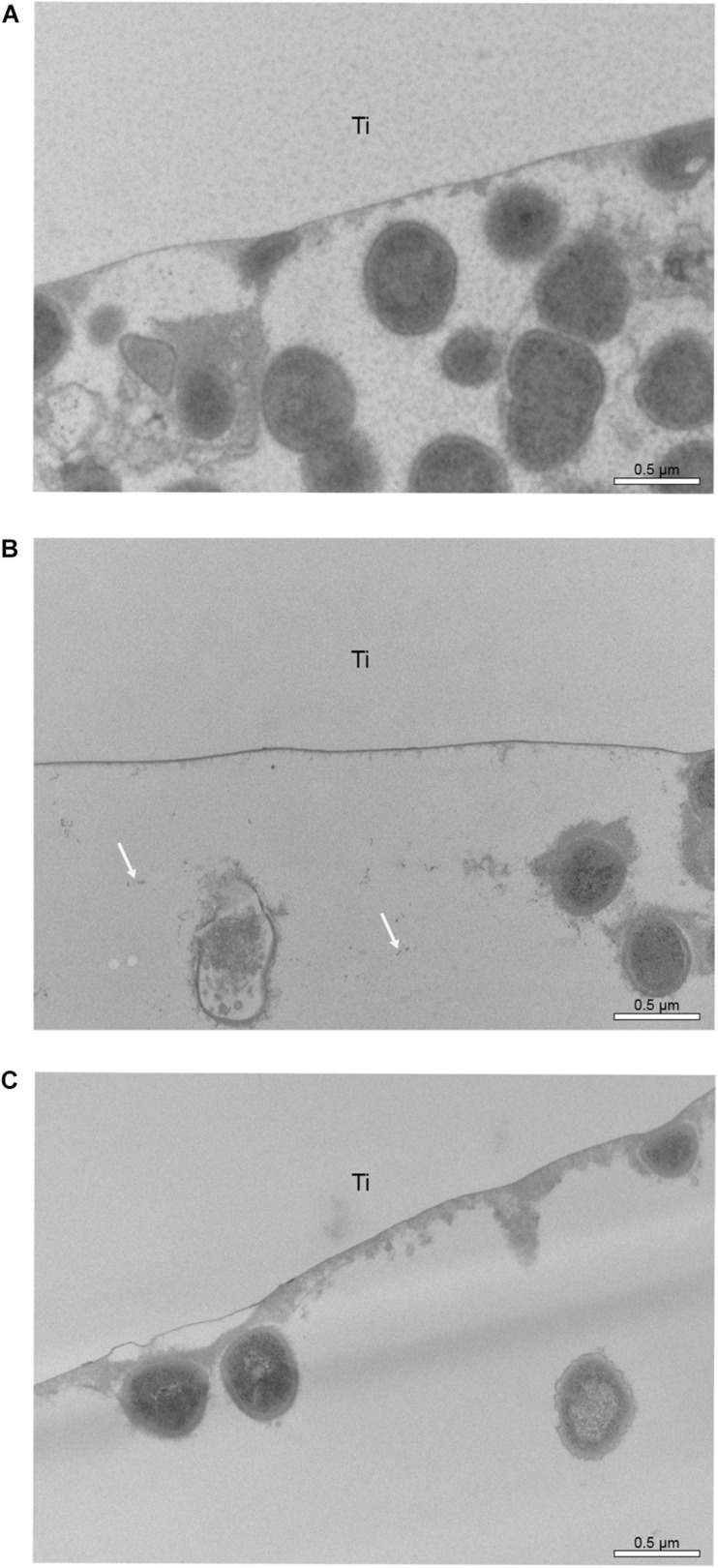
TEM micrographs of the 48-hour biofilm at 30,000-fold magnifications found on polished Ti samples after rinsing with water **(A)**, hydroxyapatite nanoparticles-based solution **(B)** and chlorhexidine **(C)**. White arrows on micrograph “b” point to probable hydroxyapatite nanoparticles. Ti indicates the former titanium specimens.

**FIGURE 11:**
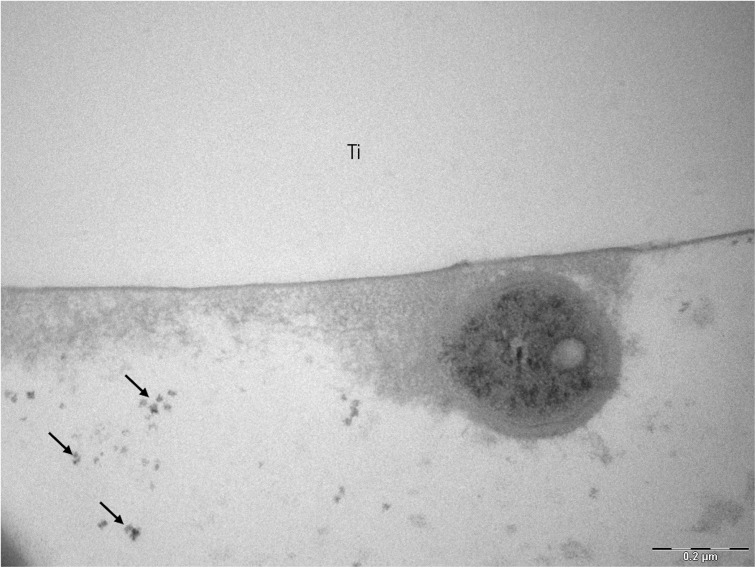
TEM analysis of a 48-hour biofilm at 68,000-fold magnification of a sample after hydroxyapatite nanoparticles-based solution rinsing. Black arrows point to hydroxyapatite nanoparticles and Ti indicates the former titanium specimen.

Transmission electron microscopic micrographs at 30.000-fold also show a higher number of bacteria in samples rinsed with water (Figure 10). On Figures 10B, 11, some small black spots scattered randomly on the samples rinsed with hydroxyapatite nanoparticles-based solution were detected.

## Discussion

This study evaluated the differences between biofilm formation under the influence of water, chlorhexidine, and an experimental hydroxyapatite nanoparticles-based solution as oral rinsing adjunct treatment on polished and non-polished titanium surfaces. Applications of the 5% watery nHAP solution could reduce the biofilm coverage of the polished titanium surfaces. In contrast to rinsing with CHX, live bacteria were present on nHAP rinsed samples, pointing to a rather biofilm modifying than an antibacterial effect.

An *in situ* experimental biofilm model was applied because of its capacity to reproduce the intraoral *in vivo* biofilm formation, which comprehend a dynamic and multifactorial process. According to Hannig and Hannig (2009) the biofilm formation process occurs differently under *in vitro* and *in vivo* conditions. *In vitro* and *in situ* models are selected according to the research question. For the topic investigated here, an *in situ* model was more suitable, since the active rinsing process cannot be performed *in vitro*. Furthermore, the antimicrobial defense mechanisms of saliva in an *in situ* model correspond to the real situation. Another advantage of the *in situ* approach is that it is possible to analyze vital biofilms with fluorescence microscopy (Hannig et al., 2007). Therefore, the *in situ* model seems to be suitable to understand the intraoral biofilm formation process properly. Intraoral removable splints were applied to proceed with the *in situ* investigation. This methodology has been used in many previous studies with excellent results (Hannig et al., 2007; Hertel et al., 2016, 2017; Kensche et al., 2017; Nobre et al., 2020). Additionally, the acrylic appliance is a convenient method for subjects, since it is easily removable during mealtimes or for oral hygiene purposes, not affecting the biofilm formation on titanium samples (Hannig, 1999). To avoid the influence of oral hygiene and diet on biofilm formation, the participants were instructed to remove the splints from the oral cavity and store them in a humid atmosphere. This avoided drying of the biofilms and minimized the influence of this interruption of the experiment. The rinsing solutions were applied in the order of their assumed effect on the biofilm: first water, then nHAP and finally chlorhexidine. In contrast to chlorhexidine, no comparable substantivity has been described for nHAP so far.

According to the SEM results, no differences in biofilm density were visible when comparing polished and non-polished samples after water rinsing. Some subjects had a slightly lower biofilm amount on polished samples, but the Ti discs presented a complex and multilayer biofilm for both types of surfaces. These small variations in biofilm formation may be due to individual factors, such as salivary flow rate, saliva composition, or dietary habits (Marsh, 2012). The biofilm could develop to advanced stages, because there were no external factors like antibacterial agents or mechanical cleaning to disturb it (Kreth et al., 2009).

Fluorescence microscopy and SEM results (Figures 3, 8) revealed a significantly denser and multilayered biofilm on the non-polished rough samples rinsed nHAP solutions when compared to the polished samples. Other *in vivo* studies have already reported this relationship between surface roughness and biofilm formation (Quirynen et al., 1990; Bollen et al., 1996; Rimondini et al., 1997; Al-Ahmad et al., 2010; Burgers et al., 2010). The increase of titanium surface roughness is directly related not only to a higher rate of biofilm formation but also to a better osseointegration (Teughels and Van Assche, 2006; do Nascimento et al., 2008). Increased roughness of titanium surfaces provides better growth of fibroblasts on the Ti substrate, establishing better osseointegration with substantial epithelial soft tissue seal around the implant (Bollen et al., 1996; Quirynen et al., 1996). However, this irregular topography facilitates bacterial adhesion and colonization (Dhir, 2013). To solve this problem, previous studies suggested a surface roughness threshold Ra value of 0.2 μm: when Ra > 0.2 μm, the biofilm formation is facilitated, whereas an Ra < 0.2 μm does not promote the biofilm formation but still supply an irregular surface proper to fibroblast fixation (Buser et al., 1991; Bollen et al., 1996; Quirynen et al., 1996). The Ra value of the non-polished titanium samples used in this study was 2 μm according to the manufacturers’ information. This is the typical roughness of the endosseous parts of many dental implants. It may explain the higher biofilm coverage of the non-polished titanium samples mainly due to their roughness, offering an attractive micro-structured surface to bacteria.

Concerning the microbial morphology, similar morphological patterns were found on polished and non-polished titanium samples in all volunteers. Coccoid shaped bacteria were present in a higher proportion, but rods could also be seen. This result agrees with the literature since gram-positive cocci and rods are the early colonizers on titanium surfaces (Steinberg et al., 1995). As observed in previous studies, there was no difference between rods and cocci proportions on both rough and smooth titanium surfaces, but a difference in thickness and biofilm density was visible (Foster and Kolenbrander, 2004; Al-Ahmad et al., 2010).

Interesting results are detected in the present study when the polished samples were analyzed. As expected, positive control samples rinsed with chlorhexidine presented a thin biofilm layer and areas without microorganism, endorsing the well-consolidated antibacterial properties of the gold standard chlorhexidine (James et al., 2017). Similar biofilm distribution was also visible in samples rinsed with the watery hydroxyapatite solution. This result may be due to a significant biofilm-formation reducing effect already shown by Kensche et al. (2017) on enamel surfaces. SEM and TEM results also confirm that hydroxyapatite nanoparticles are randomly distributed over the titanium surface, which was also demonstrated by Kensche et al. (2017). Hydroxyapatite crystallites measuring 90 to 500 nm could be identified even 12 h after the last rinse. Previous studies had also observed the hydroxyapatite particle accumulation, but on enamel surfaces and not for such a long time after the last rinsing (Hannig et al., 2013a; Kensche et al., 2017). In a recently published study using the same nHAP-based mouthrinse, it was observed that HAP nanoparticles could adhere to titanium surfaces, forming a heterogeneous layer within 2 h of intraoral exposure. In the present study the heterogeneous pattern continued to exist when the samples were removed 48 h after the beginning of the experiment (Nobre et al., 2020).

Fluorescence microscopic results of the present study demonstrated that application of CHX as a mouthwash revealed the most effective bactericidal effects, significantly reducing the vital biofilm bacteria on titanium surfaces compared to water and hydroxyapatite solutions, which is in accordance to previously published data (Hannig et al., 2013a, b; Kensche et al., 2017). This finding is related to the various chlorhexidine properties, such as broad-spectrum bacteriostatic and bactericidal effects, and substantivity, which makes it the gold standard solution on reducing bacterial vitality and biofilm formation (Hannig et al., 2013b; James et al., 2017).

Concerning biofilm coverage, nHAP was as effective as the CHX mouthrinse in decreasing biofilm formation, with no significant difference between both groups (*p* > 0.9999), demonstrating that oral rinsing with a watery solution of hydroxyapatite nanoparticles significantly reduces the number of bacteria which adhered on polished titanium surfaces. On the other hand, fluorescence microscopic images also revealed a significantly higher number of dead bacteria after treatment with CHX compared with nHAP rinsed samples (*p* = 0.0079). Similar results were already shown by Kensche et al. (2017) on enamel surfaces. The presence of live bacteria observed in the FM investigation suggests that nHAP has a rather modifying and reducing than an antibacterial effect on biofilm formation. However, when considering the present results, it is important to mention that the effect of reduced biofilm formation by the nHAP was observed on polished titanium surfaces, but not on non-polished surfaces. The less pronounced effects on rough surfaces point to a limited clinical application of nHAP in biofilm management on complex implant prosthodontics suprastructures.

According to the scarce literature in this matter, the biofilm reducing effect of hydroxyapatite is related to its particle sizes (Sakae et al., 2011; Kensche et al., 2017). This size effect facilitates the direct interaction with the bacteria, meaning that nano and sub-micron hydroxyapatite particles can interact with adhesins on the bacterial membrane, reducing the bacterial adherence (Venegas et al., 2006; Kensche et al., 2017). Kensche et al. (2017) also suggested another mechanism of action for the anti-biofilm-formation properties of hydroxyapatite particles. Hydroxyapatite particles accumulated on the pellicle-covered titanium surface could hamper the bacterial attachment to pellicle receptors blocking the interaction with cell wall adhesins from bacteria. This effect would decelerate bacterial adhesion, reducing the biofilm formation, as shown in this study (Kensche et al., 2017). In addition, it was recently observed that HAP nanoparticles can interact with the pellicle formed on titanium surface through bridge-like structures (Nobre et al., 2020). Thus, both mechanisms of nano/microparticle accumulation and receptor sites inhibition could be the reason for the higher number of dead bacteria observed by FM after nHAP solution rinsing when compared with the control water rinse.

Despite being the gold standard adjunct solution for biofilm control, long-term use of CHX is not indicated due to its well-known side effects such as teeth staining, oral mucosal erosion, and transient taste disturbance (James et al., 2017). On the other hand, after rinsing with the nHAP solution, volunteers reported an acceptable taste and no side effects during the study. Furthermore, hydroxyapatite nanoparticles mimic the dental enamel structure, and for being a biocompatible solution, side effects are unlikely to occur (Epple, 2018). Additionally, results suggested that nHAP had biofilm-formation modulating but not antimicrobial effects, therefore, the tested solution is less likely to impact on the oral cavity homeostasis. Finally, literature states that hydroxyapatite particles are dissolved in gastric fluid in case of ingestion, not being harmful to the human body (Kensche et al., 2017).

The small number of subjects involved in the present study was a limitation of this investigation. The complexity of the *in situ* methodology and the electron microscopic techniques used for biofilm analyses were the reasons to select a small number of volunteers, such as in previous studies with similar methods (Al-Ahmad et al., 2009; Jung et al., 2010; Kensche et al., 2017).

Another interesting question is the influence of nHAP on the microbial diversity of oral biofilms. Studies on this issue would also require a higher number of volunteers. Depending on the methodology, culture or sequencing techniques, sufficient biofilm mass would have to be generated. This should also be a topic of a follow up study.

The results of this investigation indicated that the experimental 5% solution of pure hydroxyapatite nanoparticles reduced *in situ* oral biofilm formation on titanium surfaces, representing a novel bioinspired approach for biofilm management without altering bacterial viability. Additionally, independent from the rinsing solution used, a thicker and multilayer biofilm coverage was present on the non-polished samples. Thus, titanium surface morphology reveals a strong impact on bacterial colonization.

## Data Availability Statement

The raw data supporting the conclusions of this article will be made available by the authors, without undue reservation.

## Ethics Statement

The studies involving human participants were reviewed and approved by Medical Ethics Committee of the Medical Association of Saarland, Germany. The participants provided their written informed consent to participate in this study.

## Author Contributions

CN and MH: conceptualization, methodology, project administration, and visualization. CN, NP, and BK: formal analysis. MH: funding acquisition and supervision. CN: investigation. SR and MH: resources. CN and BK: writing – original draft. CN, SR, and MH: writing – review and editing. All authors have read and agreed to the published version of the manuscript.

## Conflict of Interest

The authors declare that the research was conducted in the absence of any commercial or financial relationships that could be construed as a potential conflict of interest.
